# Absence of modulatory action on haptic height perception with musical pitch

**DOI:** 10.3389/fpsyg.2015.01369

**Published:** 2015-09-11

**Authors:** Michele Geronazzo, Federico Avanzini, Massimo Grassi

**Affiliations:** ^1^Department of Information Engineering, University of PadovaPadova, Italy; ^2^Department of General Psychology, University of PadovaPadova, Italy

**Keywords:** sensory integration, height estimation, multimodal virtual environment, haptic virtual objects, musical pitch

## Abstract

Although acoustic frequency is not a spatial property of physical objects, in common language, pitch, i.e., the psychological correlated of frequency, is often labeled spatially (i.e., “high in pitch” or “low in pitch”). Pitch-height is known to modulate (and interact with) the response of participants when they are asked to judge spatial properties of non-auditory stimuli (e.g., visual) in a variety of behavioral tasks. In the current study we investigated whether the modulatory action of pitch-height extended to the haptic estimation of height of a virtual step. We implemented a HW/SW setup which is able to render virtual 3D objects (stair-steps) haptically through a PHANTOM device, and to provide real-time continuous auditory feedback depending on the user interaction with the object. The haptic exploration was associated with a sinusoidal tone whose pitch varied as a function of the interaction point's height within (i) a narrower and (ii) a wider pitch range, or (iii) a random pitch variation acting as a control audio condition. Explorations were also performed with no sound (haptic only). Participants were instructed to explore the virtual step freely, and to communicate height estimation by opening their thumb and index finger to mimic the step riser height, or verbally by reporting the height in centimeters of the step riser. We analyzed the role of musical expertise by dividing participants into non-musicians and musicians. Results showed no effects of musical pitch on high-realistic haptic feedback. Overall there is no difference between the two groups in the proposed multimodal conditions. Additionally, we observed a different haptic response distribution between musicians and non-musicians when estimations of the auditory conditions are matched with estimations in the no sound condition.

## Introduction

The classic approach to perception investigates one sense at time (Fechner, 1889). This approach is useful to understand how single senses work, but it does not take into account that perception is intimately a multimodal process. In the last three decades, there has been a growing interest in investigating perception as a multimodal process. The multimodal approach to perception has led to questions that are still largely unanswered, such as how the percepts from the different senses are integrated to form a coherent picture of the sensory world (see Calvert et al., 2004, for an overview).

In some cases, the way percepts are bound is relatively well-known such as when we gather the same information through different senses, for example, when we estimate the size of one object with our eyes and with our hands. According to Ernst and Banks (2002, but see also Ernst and Bülthoff, 2004) in such circumstances the multimodal size-estimate of the object can be modeled as the weighted sum of the uni-modal size-estimates. This model accounts for various phenomena, also when using senses other than touch and vision (e.g., Alais and Burr, 2003; Grassi and Pavan, 2012; see Burr and Alais, 2006 for a quick overview).

However, interactions occur also when the information gathered by different senses is linked in subtler ways. For example, although acoustic frequency is not a spatial property of physical objects, several experiments report a correspondence between pitch and physical size (Spence, 2011). Gallace and Spence (2006) asked participants to perform a speeded visual size discrimination task and judge whether a variable-sized disk was larger or smaller than a reference one. A task-irrelevant sound that was either congruent with the relative size of the disk (e.g., a low-frequency sound with a larger disk) or incongruent with it (e.g., a low-frequency sound with a smaller disk) was presented. Reaction times were shorter in the congruent condition than in the incongruent condition. A similar result was also observed by Evans and Treisman (2010), who additionally observed that the modulatory action of pitch was not limited to the size of the stimulus but extended to its vertical position in the visual display. In common language, pitch is often labeled spatially (i.e., “high in pitch” or “low in pitch”). Almost a century ago, Pratt (1930) observed that high pitched tones are phenomenologically higher in space than low pitched tones. Rusconi et al. (2005, 2006) showed that this spatial connotation interacts with motor action so that, when we are asked to respond quickly whether a pitch is high or low in comparison to a reference pitch, we are faster if the response is coherent with the spatial position of the response key (e.g., the response is “high” and the response key is in the upper part of the keyboard), rather than vice-versa. Noticeably, the association between pitch-height and spatial height has also been observed in children (Walker et al., 2010; Nava et al., 2015).

Overall, the general tendency to associate high/low-pitched sounds with small/large dimensions has a counterpart in the physical world. In impact sounds, for example, large objects tend to produce sounds that are lower in frequency than smaller objects (Grassi, 2005; Grassi et al., 2013). In addition, another fact linking frequency to spatial properties is that high-frequency sounds tend to originate from elevated sound sources (Parise et al., 2014). In both cases, although acoustic frequency has not, *per se*, any spatial property, the continuous perception of events where frequency is associated with size (or height) in a systematic fashion could be learned by humans.

The modulatory action of sound is not limited to vision. Castiello et al. (2010) recorded sounds produced by the fingers while grasping a ball covered in various materials. In a successive session, sounds were delivered to participants before or following the initiation of reach-to-grasp movements toward the ball. Movement kinematics were measured under three conditions: (i) congruent, in which the presented contact sound and the contact sound elicited by the to-be-grasped stimulus corresponded; (ii) incongruent, in which the two sounds were different; (iii) control, in which a synthetic sound, not associated with a real event, was presented. Authors observed facilitations (i.e., lower grasping time) for congruent trials and interference for incongruent trials. Similarly, Sedda et al. (2011) observed that the impact sound of an object (either small or large) placed on a table modulated the grip aperture of blind participants that were are asked to grasp the object. The grip aperture was larger when the sound was rich in low frequency components (i.e., compatible with a larger object) than when the sound was rich in high frequency components (i.e., compatible with a smaller object).

Research in multimodal perception provides the ground for the design of multimodal interfaces and virtual environments. It has been long recognized that properly designed haptic (Srinivasan and Basdogan, 1997) and auditory (Hahn et al., 1998) displays can provide greater immersion in a virtual environment than a high-fidelity visual display alone. Audio-haptic interaction is particularly interesting for several applications involving interaction with virtual objects and environments, particularly interfaces for non-sighted users. Perceptual distortions occurring in virtual environments have been the subject of several studies. As an example (related to vision), numerous results show that distance is compressed in immersive virtual environments presented via head mounted display systems, relative to in the real world (Messing and Durgin, 2005). Regarding the haptic perception of size and lengths of virtual objects, it is known that distortions can occur due to limitation in the hardware and in the haptic rendering: Tan and coworkers (Choi et al., 2005) showed that, when using a stylus-based haptic device, subjective thresholds in surface-height discrimination depend on the surface stiffness rendered by the device, and that alterations of the surface stiffness can induce reversals of perceived heights of pairs of virtual steps. Wydoodt et al. (2006) showed that disruptions to force cues in the haptic feedback (e.g., elastic or viscous opposition) can produce under- or over-estimations of object lengths. Some studies also suggest that, being the interaction mediated by the device (e.g., the stylus), different devices can produce different percepts (Penn et al., 2000).

Given that audition modulates motor actions and that pitch-height modulates size and height perception of the objects, in this study we investigated whether pitch could modulate the size estimate of objects that were explored haptically in a virtual environment. During the experiment, participants were asked to explore the height of various virtual steps by means of a PHANTOM^1^ device. After each exploration, the participant estimated the step riser height by placing the open thumb and index fingers on a tablet pc that recorded the aperture of the fingers. Successively, the participant provided a verbal, metric estimate. The experiment used two experimental conditions and two control conditions. In the experimental conditions, while moving the PHANTOM stylus along the step riser the participant could listen to a tone, whose pitch increased as the stylus moved upward along the step riser and decreased along the opposite direction. The pitch range of the tone was a monotonic function of the step riser height, with shorter step risers associated with narrower pitch ranges. In particular, two mappings were used with one of the two mappings having a wider pitch range. The first control condition served to understand whether the presence of sound *per se* could affect the size estimate of the step riser. In this condition the pitch of the accompanying sound changed in a random fashion with the stylus position along the step. In the second control condition the exploration of the virtual step was accompanied by no sound. The hypothesis behind the experiment was that the participant's estimate of the step riser height could be modulated by mapping of the two experimental conditions, namely, larger estimates for the wider frequency range and smaller estimates for the narrower frequency range. No predictions were made for estimates gathered in the control conditions nor for the relationship between these estimates and those gathered in the experimental conditions.

However, the data presented in the remainder or the paper show that this hypothesis was not confirmed by experimental results. Participants were very accurate in their estimates in all experimental conditions, including the control condition with no auditory feedback, and they did not produce larger estimates for stimuli with larger frequency sweeps. The only exception to these results was found for the group of participants with musical expertise, for which a statistically significant effect of sound with respect to non-musicians was observed in the exploration task. Section Materials and Methods describes in detail the experimental protocol and setup, while Section Results summarizes our analysis on the collected data. Section General Discussion provides a general discussion of the experimental results, as well as motivations for further research on this topic.

## Materials and methods

### Subjects and apparatus

Twenty-four participants (7 males and 17 females), aged between 20 and 24 (mean = 21, *SD* = 0.9), took part to the experiment. All of them signed the consent form and a demographics questionnaire. The experimental procedure described here was in accordance with the Declaration of Helsinki (Edition 2013). According with the Edinburgh Handedness Inventory by Oldfield (1971), 19 participants were right-handed and 6 were ambidextrous. All participants self-reported normal hearing and no impairment in limb movements. They were students and apprentices of the University of Padova and had no knowledge nor experience of haptic force-feedback devices.

**Figure 1:**
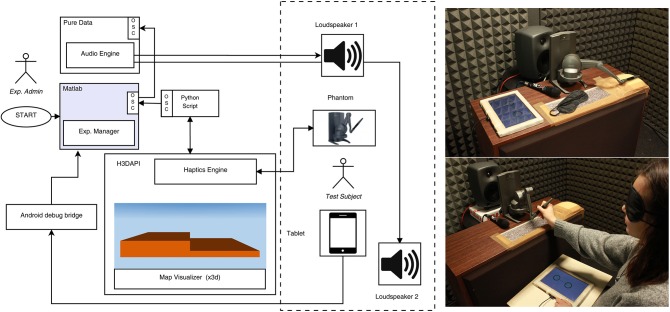
**A simplified scheme of the experimental setup and spatial arrangement in the silent booth**.

The experimental setup and the interactive multimodal virtual environment are outlined in Figure 1. The experiment was carried out in a sound proof booth. A computer running MATLAB acted as the control unit. The computer was connected to a Sensable PHANTOM Desktop device which is able to render a high realistic haptic scene with a nominal position resolution of ~10^−5^ m. A Motu 896 mk3 sound card transmitted the acoustic feedback and notification sounds to two separate audio out-channels (Genelec 8030A loudspeakers). H3DAPI^2^ (an open source platform using OpenGL and X3D^3^ with haptics in one unified scene graph) and Pure Data^4^ (an open source real-time environment for audio processing) rendered the multimodal feedback exchanging messages though Open Sound Control (OSC). An *ad-hoc* software was developed for the Android Tablet Samsung Galaxy Tab 10.1 in order to collect participant responses.

### Stimuli

Participants were asked to estimate the heights of *N* = 8 virtual step risers linearly spaced on a logarithmic scale between *h*_1_= 2 cm and *h*_*N*_= 10 cm. The following equation describes the height of the i-th step rise:

$${\text{h}}_{i}=h_{1}*{\left(\frac{h_{N}}{h_{1}}\right)}^{\frac{i-1}{N-1}}.$$

All the steps spanned a 22 × 22 = 484 cm^2^ horizontal square area (see Figure 1) in order to guarantee a sufficiently large workspace. Each step riser lied in the yz-plane of the virtual scene and in the midsagittal plane related to participant posture. The upper tread of the step riser lied at the left of the yz-plane facilitating right-handed participants in the exploration. Normalized static and dynamic friction coefficients were set to 0.1 and 0.4 respectively. The normalized stiffness was set to 1 so as to render an impenetrable virtual stair-step without causing the device to become unstable.

Upon collision of the cursor with the step rise, three multimodal feedback and a uni-modal feedback were provided. The latter acted as control condition for the experiment being the haptic rendering alone. The remaining multimodal conditions added the auditory modality providing acoustic information based on the y-coordinate of the *haptic interaction point* (HIP).

The mapping between y-coordinate and the frequency, *f*, of a sine wave produced a dynamically modulated sinusoid while the participant explored the virtual step, generating a continuous interaction with the surface. This mapping was defined through three parameters:

- *f*_*min*_: the lowest frequency associated to ground level;
- Δ*f*: the frequency range spanned above *f*_*min*_ leading to the maximum frequency associated with the upper tread in the form of *f*_*max*_ = *f*_*min*_ + Δ*f*;
- The mapping strategy: the function that maps HIP to the frequency domain [*f*_*min*_, *f*_*max*_].

A further distinction was made between informative or non informative audio feedback, leading to two different mapping strategies:

- Frequency following the standard Western chromatic scale:
  $$f=f_{min}\;*\;{\left(\frac{f_{max}}{f_{min}}\right)}^{\hat{h}}$$
  where ĥ = *h/h*_*N*_ denotes the normalized y-coordinate of HIP in the range of [0, 1], corresponding to the range 2 cm to 10 cm in the experiment.
- Random frequency according to the following equation:
  $$\begin{array}{l} f=f_{a}^{rand}+h^{n,\;\left[a,b\right]}\;*\;\left(f_{b}^{rand}-f_{a}^{rand}\right),\\ \;with\left[a,\;b\right]⇐\left\lfloor h\right\rfloor ,\;h^{n,\;\left[a,\;b\right]}=h-\left\lfloor h\right\rfloor , \end{array}$$
  which is a linear interpolation of *f* between $f_{a}^{rand}$ and $f_{b}^{rand}$ randomly computed for every centimeter in *h*, except for the ground level where the interpolation value is always C4 at 261.626 Hz. In practice, this mapping produces sinusoid frequencies that always correspond to C4 at the ground level and vary pseudo-randomly with the y-coordinate of HIP, with no recognizable functional dependence.

**Figure 2:**
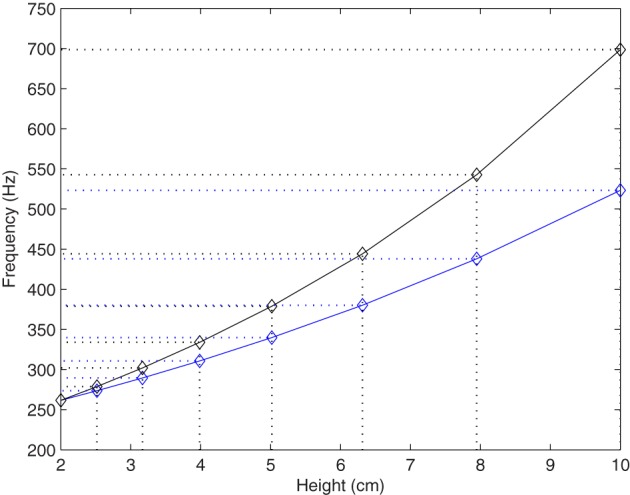
**Height to frequency mapping**. Bi-modal feedback conditions “pitch 1” (blue line) and “pitch 2” (black line).

The parameters choice for auditory feedback resulted in four experimental conditions which are summarized in Table 1. Ground level (*h* = 0) was always associated with *f*_*min*_ = C4 at 261.626 Hz. In the informative bi-modal conditions (*pitch 1* and *pitch 2*), **Δ*f*** is equal to 1-octave interval and 1-octave plus a perfect 4th musical interval, respectively: the mapping between frequency pitch and *h* is depicted in Figure 2. In the non-informative bi-modal condition (*random pitch*), $f_{a,\;b}^{rand}$ < C5.

**Table 1:** **Multimodal experimental conditions**.

**Name**	**Type**	**Mapping strategy**	***f_*min*_***	***f_*max*_***	***Δf***
Silence	Uni-modal (control condition 1)	–	–	–	–
Random pitch	Bi-modal (control condition 2)	Random frequency unrelated to height	C4 (261.626 Hz)	< C5 (523.251 Hz)	–
Pitch 1	Bi-modal	Standard Western chromatic scale	C4 (261.626 Hz)	C5 (523.251 Hz)	12 semitons (261.626 Hz)
Pitch 2	Bi-modal	Standard Western chromatic scale	C4 (261.626 Hz)	F5 (698.456 Hz)	17 semitons (436.830 Hz)

### Procedure

**Figure 3:**
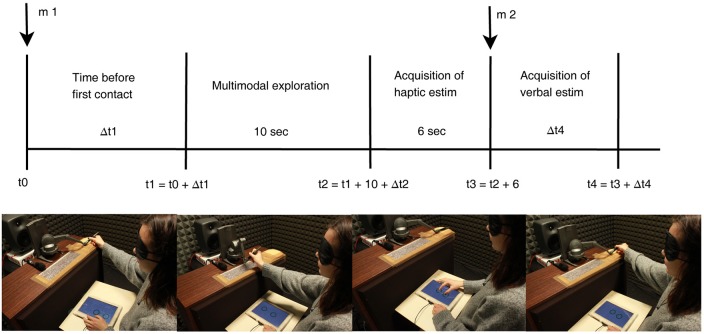
**Time-line of a single trial**. Four pictures depict: begin of a trial, exploration phase, haptic estimation phase, and verbal estimation phase with return to starting position, respectively. At instants t0 and t3, two audio messages are played in Loudspeaker 2 signaling trial begin, m1, and the end of available time for haptic estimation, m2. Δt2 and Δt4 depict participant-dependent time interval.

Before being blindfolded, participants were informed about the use of the stylus for exploring virtual steps and about the experimental phases of each trials in order to perform the estimation task. Figure 3 depicts the time-line of a single trial. At t0 instant, a “beep” audio message, m1, signaled the beginning of the trial and the participant could move from the starting position at the far right of the workspace toward the step; once they reached the step-rise, a 10-s timer counted the available time for the exploration phase whereupon the virtual object disappeared. Starting from this moment, once the participants came in contact with the touchscreen, a 6-s period was provided in order to haptically estimate the step rise height placing their right-hand fingers on the tablet screen which laid on the participant lap. Participants were free to move their thumb and index finger on the touch-screen in order to estimate height as the distance between these two fingers. At the end of the available time, the last final contact of the fingers was acquired and considered for the trial. At the same instant, a bell audio message, m2, marked that the participant had to provide also the verbal estimation. Participants were instructed to be accurate to 1 mm in their verbal estimation; the experimenter took note of participant estimations by listening to them through a microphone placed in the sound proof booth. Once the verbal estimation was collected, the stylus was placed in the starting area and the experiment moved to the next trial.

No feedback concerning the accuracy was given to the participant. The role of the auditory height-dependent sinusoidal feedback was not explained or commented. At the end of the trials participants were guided out of the sound proof booth and were asked to answer to a post-experimental questionnaire.

The 32 stimuli (8 heights × 4 feedback conditions) were presented with 2 repetitions leading to a total of 64 trials. The order of stimulus presentation was divided in two blocks: the first block presented all 32 stimuli and the second block presented their repetitions. In each block, trial order was randomized with the only constraint that consecutive trials had not the same height^5^.

### Measures and data analysis

The evaluation of sensory integration indicators were made starting from haptic height estimations in centimeters and verbal height estimations in centimeters. In addition, both the haptic and the verbal estimations were transformed into percentage estimates (**E1**) as follow:

$$E1.\;percentage\;error=\frac{subjective\;estimate-actual\;height}{actual\;height}*100$$

where subjective estimate is the participant estimate, either haptic or verbal, in centimeters and actual height is the actual height of the step riser in centimeters.

In order to take into account the subjective height of the step riser in the control-silent condition, a further perceptual error metric (**E2**) was introduced and estimations were scaled to subjective unimodal (i.e., silent) haptic/verbal estimation. The unimodal silent condition acts as “*ground truth”* in order to reveal possible auditory integration effects. **E2** was calculated as follow:

$$E2.\;perceptual\;error=subjective\;estimate-height_{silence}$$

where subjective estimate is the subjective estimate in centimeters in the non-silent condition (either haptic or verbal) and height-silence is the subjective estimate in centimeters in the silent condition.

In addition to the above measures, at the end of the experiment participants answered to the following short questionnaire concerning their self-reported level of sensory integration and of musical expertise:

**Q1**. Indicate to which extent virtual objects were realistic;
**Q2**. Evaluate to which extent the auditory feedback helped your estimates;
**Q3**. Evaluate to which extent height estimates were accurate;
**Q4**. Do you play a musical instrument;
**Q5**. Indicate time spent in weekly musical practice.

Responses to questions **Q1, Q2, Q3** were given on a visual analog scale (VAS) [0 = not at all, 10 = very much].

Because musicians are more sensitive to the association between pitch and height than non-musicians (Rusconi et al., 2006) participants were divided in two groups according to their level of musical expertise and practice. Based on the yes/no responses to question **Q4** in the questionnaire, two groups of 14 non-musicians and 10 musicians were created.

Haptic height estimations, verbal height estimations and error metrics **E1** and **E2** (both haptic and verbal) were subjected to 4 (or 3 auditory feedback conditions) by 8 (step riser heights) analysis of variance (ANOVA) with one between factor (group of musical expertise: musicians vs non-musicians). Preliminary analyses of gaussianity, sphericity were calculated on each factor combination by means of the Kolmogorov-Smirnov test and the Mauchly test. Overall, gaussianity and homoscedasticity were not violated. In the case of sphericity violations the Greenhouse-Geisser corrected *p*-value was selected^6^. We analyzed the data with three different alpha levels using Bonferroni algorithm in order to provide a broader view of our results, ranging from the less to the more conservative: 0.05 (no adjustment), 0.05/3, and 0.05/6 (Wright, 1992; Bender and Lange, 2001). In the second case, the three global ANOVAs on haptic estimations (or verbal estimations) were corrected separately, while in the third case all six global ANOVAs belonged to a single family. In sake of simplicity, in the Results section, we reported the unadjusted *p*-values with specific comments where differences in statistical power arose. Furthermore, we calculated descriptive statistics for the answers to the questionnaire and three independent samples *t*-tests to assess possible differences between musicians and non-musicians in the answers to **Q1**, **Q2**, and **Q3**.

## Results

**Figure 4:**
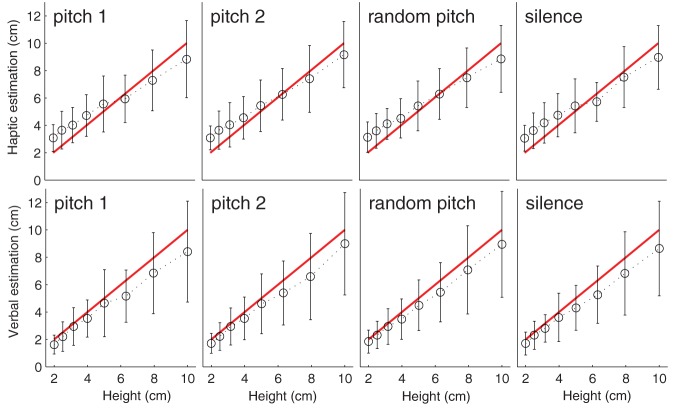
**Height estimations**. Mean and standard deviation (error-bars) of haptic (first row) and verbal (second row) estimations for each of the four feedback conditions and eight heights. Red lines denote actual heights of the step-riser.

Figure 4 depicts haptic and verbal height estimates averaged separately for step riser height and feedback condition. Overall, the haptic size estimation was not statistically different between the two groups, *F*_(1, 22)_ = 0.03, *p* = 0.86, but the haptic size estimation increased as a function of the step-riser height, *F*_(7, 154)_ = 230.4, *p* < 0.0001. However, the group interaction with step-riser height was not significant: *F*_(7, 154)_ = 0.594, *p* = 0.76. The haptic size estimation was not modulated by the auditory feedback: *F*_(3, 66)_ = 0.31, *p* = 0.82. The group interactions with auditory feedback was also not significant: *F*_(3, 66)_ = 1.31, *p* = 0.28. The two ways interaction between step-riser height and auditory feedback was not significant: *F*_(21, 462)_ = 1.09, *p* = 0.35. Moreover, the three way interaction was not significant: *F*_(21, 462)_ = 1.29, *p* = 0.18.

Similar, results were observed for verbal estimation. Verbal estimates were not statistically different between the two groups after alpha level adjustment, *F*_(1, 22)_ = 4.64, *p* = 0.04, (< 0.05, >0.02, and >0.008), however they increased as a function of step-riser height, *F*_(7, 154)_ = 127.7, *p* < 0.0001. Moreover, the group interaction with step-riser height was significant: *F*_(7, 154)_ = 2.96, *p* = 0.006. The auditory feedback did not modulate the verbal response, *F*_(3, 66)_ = 1.82, *p* = 0.15 and the group interaction was not significant: *F*_(3, 66)_ = 0.33, *p* = 0.81. The two ways interaction between step-riser height and auditory feedback was not significant: *F*_(21, 462)_ = 1.02, *p* = 0.44. Moreover, the three way interaction was not significant: *F*_(21, 462)_ = 0.29, *p* = 0.99.

**Figure 5:**
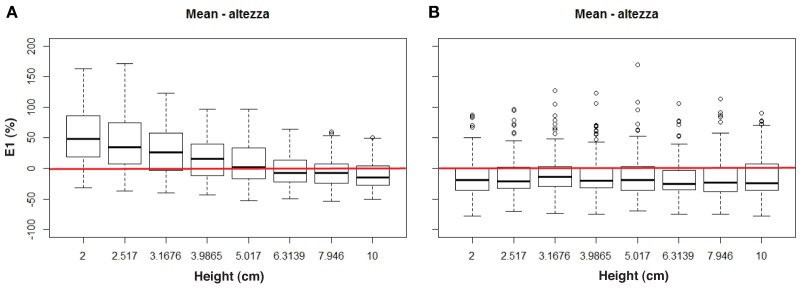
**Mean haptic percentage error (A) and mean verbal percentage error (B) for each height, grouping all feedback conditions**.

In order to assess how the error in the height estimation changed as a function of the step riser height and feedback condition, haptic and verbal estimates were transformed in percentages of under- or over-estimation of haptic and verbal height, i.e., **haptic-E1** and **verbal-E1**. The **haptic-E1** was not statistically different between the two groups, *F*_(1, 22)_ = 0.003, *p* = 0.96. It decreased as a function of the step-riser height, *F*_(7, 154)_ = 75.4, *p* < 0.0001 because participants overestimated the size of smaller step riser but estimated veridically the height of larger step riser (see Figure 5). Moreover, the interaction between group and step-riser height was not statistically significant, *F*_(7, 154)_ = 0.54, *p* = 0.80. **Haptic-E1** was not modulated by the auditory feedback: *F*_(3, 66)_ = 0.08, *p* = 0.97 and the interaction with the group was not significant: *F*_(3, 66)_ = 0.35, *p* = 0.78. The two ways interaction between step-riser height and auditory feedback was not significant: *F*_(21, 462)_ = 0.66, *p* = 0.87. Moreover, the three way interaction was not significant: *F*_(21, 462)_ = 1.32, *p* = 0.15.

Different results were observed for the **verbal-E1**. **Verbal-E1** did not change as a function of the group after alpha level adjustment, *F*_(1, 22)_ = 4.74, *p* = 0.04 (< 0.05, >0.02, and >0.008), nor of the step riser height as participant's estimate showed a constant underestimation of the step riser height: *F*_(7, 154)_ = 1.29, *p* = 0.26. Additionally, the interaction between these two factors was not statistically different, *F*_(7, 154)_ = 0.41, *p* = 0.89. The auditory feedback did not modulate the **verbal-E1**, *F*_(3, 66)_ = 2.29, *p* = 0.08, nor the interaction with the group was significant: *F*_(3, 66)_ = 0.17, *p* = 0.92. The two ways interaction between step-riser height and auditory feedback was not significant: *F*_(21, 462)_ = 0.93, *p* = 0.54. Moreover, the three way interaction was not significant: *F*_(21, 462)_ = 0.59, *p* = 0.93.

We conducted a further analysis (i.e., a 3 auditory conditions by 8 step riser heights ANOVA with one between factor) on **haptic-** and **verbal-E2**. Results for **haptic-E2** confirmed that the group had not a significant contribution in the estimation with bi-modal feedback, *F*_(1, 22)_ = 3.04, *p* = 0.09, nor the height had not a significant contribution: *F*_(7, 154)_ = 1.62, *p* = 0.13. Interestingly, the interaction between these two factors was statistically different except for the most conservative alpha level adjustment, *F*_(7, 154)_ = 4.12, *p* = 0.0157 (< 0.05, < 0.02, and >0.008), reporting a difference in height estimations between the two groups with auditory feedback. However, no statistical significances were found among auditory conditions, *F*_(2, 44)_ = 0.42, *p* = 0.66, and the interaction between group and condition, *F*_(2, 44)_ = 0.46, *p* = 0.63. The two ways interaction between step-riser height and auditory feedback was not significant: *F*_(14, 308)_ = 0.82, *p* = 0.65. Moreover, the three way interaction was not significant: *F*_(14, 308)_= 0.65, *p* = 0.82.

The same overall ANOVA was computed for **verbal-E2**, reporting no significant statistical effects. The two groups did not differentiate, *F*_(1, 22)_ = 0.34, *p* = 0.57. Neither the step-riser height nor the interaction between height and group were statistical significant, *F*_(7, 154)_ = 0.67, *p* = 0.69 and *F*_(7, 154)_ = 0.49, *p* = 0.84, respectively. Again, results reported a non significant effect of the auditory condition, *F*_(2, 44)_ = 2.96, *p* = 0.06 and a non significant interaction with the group, *F*_(2, 44)_ = 0.31, *p* = 0.73. The interaction between feedback condition and height was not significant, *F*_(14, 308)_ = 1.15, *p* = 0.31, nor the three way interaction, *F*_(14, 308)_ = 0.22, *p* = 0.99.

**Figure 6:**
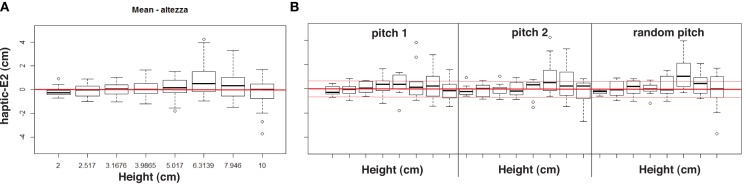
**Results for musicians**. Mean haptic error **(A)** for each height, grouping all feedback conditions. Mean haptic percentage error for each bi-modal condition **(B)**; red thin lines indicate the ±0.66 cm threshold.

**Figure 7:**
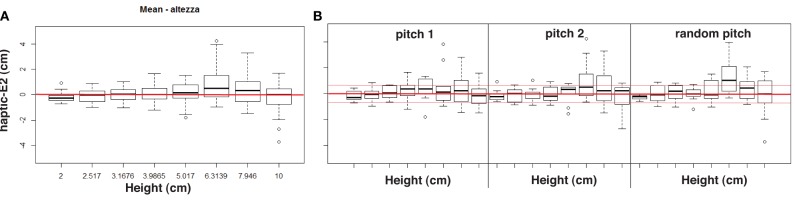
**Results for non-musicians**. Mean haptic error **(A)** for each height, grouping all feedback conditions. Mean haptic percentage error for each bi-modal condition **(B)**; red thin lines indicate the ±0.66 cm threshold.

Among the two measures, **E2** and in particular **haptic-E2**, should be the most sensitive to auditory feedback as it takes into account the subjective estimate of the participant in the control (i.e., *silence*) condition, evaluating the contribution of sound only. Having found a significant effect in the interaction between group and step-riser height for **haptic-E2**, the aim of the following analysis is to provide a behavioral interpretation of the influence of sound for the two groups. Figures 6, 7 depict **haptic-E2** values for the two groups; in particular, trials with larger errors, i.e., near the tails of the normal distributions, were defined in terms of over- and under- estimations for each auditory condition, allowing the use of wide ranges in the data. For the sake of this analysis, over- and under-estimations were defined as estimates that exceeded (positively or negatively) the average value among unsigned **haptic-E2** estimates across trials.^7^ This average value is 0.66, therefore trials with **haptic-E2** > 0.66 were considered over-estimations, whereas those with **haptic-E2** < −0.66 were considered as under-estimations.

**Figure 8:**
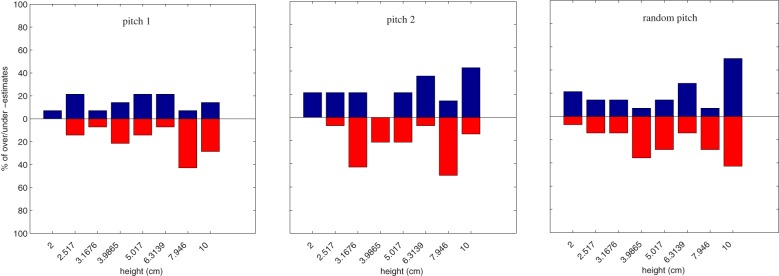
**Percentage of over estimations (blue bars, 0–100% upward) and under estimations (red bars, 0–100% downward) over all non-musicians for each height**. From left to right, pitch1, pitch2, and random pitch conditions.

**Figure 9:**
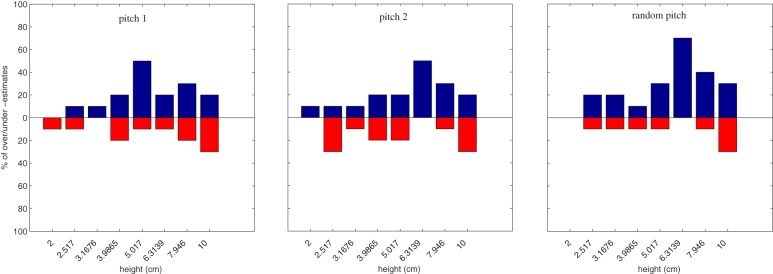
**Percentage of over estimations (blue bars, 0–100% upward) and under estimations (red bars, 0–100% downward) over all musicians for each height**. From left to right, pitch1, pitch2, and random pitch conditions.

Figures 8, 9 depict percentages of over- and under-estimations computed in this way for the two groups of non-musicians and musicians separately. Each blue (red) bar denotes the percentage of over- (under-) estimations among all available estimations with equal height and condition into the same group (musicians vs. non-musicians).

**Figure 10:**
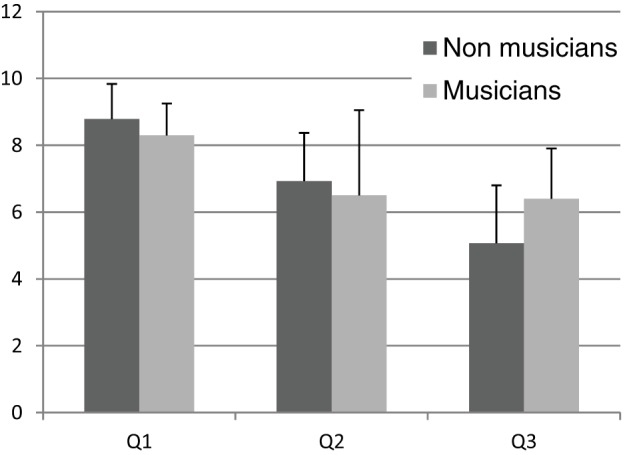
**Post-experimental questionnaire: Q1, Q2, and Q3**.

Responses to the questionnaire helped to understand how participants evaluated their performance (see Figure 10 for qualitative results in **Q1**, **Q2**, and **Q3**). Responses revealed that participants judged virtual steps with high realism (**Q1**, mean score 8.79 ± 1.05 for non-musicians and 8.3 ± 0.95 for musicians) and an independent sample *t*-test between the two groups did not show any significant difference, *t*_(20)_ = 1.18, *p* = 0.25. Questionnaire responses to perceived usefulness of the auditory feedback (**Q2**) revealed no statistical difference between the two groups, *t*_(13)_ = 0.48, *p* = 0.64. Non-musicians and musicians reported mean scores of 6.93 ± 1.44 and 6.5 ± 2.55, respectively (on a 10-points VAD scale). This result suggests that non-musicians attributed a role to audio within a 1½-point consensus. On the other hand, musicians attributed weaker role to audio with a large disagreement of 2½-points. Moreover, participants slightly differed in self-reported precision (**Q3**), *t*_(21)_ = 2.00, *p* = 0.06. Non-musicians rated their estimates as rather inaccurate (mean score 5.07 ± 1.73 on a 10-points VAD scale), whereas musicians were more self-confident (mean score 6.40 ± 1.51 on a 10-points VAD scale).

## General discussion

The hypothesis of this study was that, because pitch height often interacts with the response of participants in behavioral experiments (e.g., Gallace and Spence, 2006; Evans and Treisman, 2010) with particular emphasis on further distinctions based on musical expertise (Rusconi et al., 2006), auditory feedback would modulate haptic perception of the height of a step. More precisely, we expected larger estimates of step-riser heights for wider frequency sweeps (i.e., condition *pitch2*) than for narrower frequency sweeps (i.e., condition *pitch1*), while no predictions were made for the random pitch and the no sound condition. This hypothesis was not confirmed by experimental results: we did not observe an effect of auditory pitch on step height, i.e., participants did not produce larger estimates for stimuli with larger frequency sweeps.

Overall, results showed that participants' estimations (both haptic and verbal) increased monotonically as a function of the step riser height. When haptic estimations were converted into percentages of under/over-estimations (i.e., **haptic-E1**), participants exhibited a tendency in over-estimating heights smaller than 5 cm whereas larger step riser heights were estimated almost veridically (see Figure 5A). The over-estimation of the small steps can be explained. The participant evaluated the step rise by opening the thumb and the index finger. The tablet device captured this motor action. It is possible that the tablet device introduces a constant error. A constant error, of any magnitude, would affect largely the smallest estimates and negligibly the larger estimates. By the same token, a constant motor error of the participant when opening the fingers (i.e., such as a little tremor) would have exactly the same effect on the estimates. For this reason, verbal estimates (**verbal-E1)** did not show a similar pattern of results. Verbal estimates were characterized by a small, constant underestimation for all the heights (see Figure 5B) confirming preliminary results from Geronazzo et al. (2013).

The second result is that neither type of estimate (haptic and verbal) was significantly affected by the auditory feedback accompanying the exploration of the virtual step. Although according to the questionnaire participants found the auditory feedback useful, this assertion contrasts with the behavioral results that show no significant difference between the results of the *silence* condition and the two informative bimodal conditions (*pitch1* and *pitch2*). In addition, the presence of the sound *per se*, with no informative content (e.g., the random pitch condition), did also not affect the estimation.

The main reason why sound did not affect haptic and verbal estimates is possibly that haptic estimate of size is so robust that the modulatory action of sound does simply not occur or, in alternative, might occur only when the haptic estimate is impaired such as predicted by the model of Ernst and colleagues (Ernst and Banks, 2002; Ernst and Bülthoff, 2004). This explanation is supported by the fact that participants were very accurate in their estimates in all conditions, including the *silence* condition. In fact, although previous literature provides many evidences supporting the hypothesis that auditory pitch interacts with spatial perception, the majority of these evidences come from vision and in speeded tasks such a reaction time tasks. In addition, it is also possible that some participants simply ignored the sound at all (see high variability for **Q2** in Figure 10).

There is however one exception to the results discussed so far. When considering the group factor of non-musicians and musicians, a statistically significant effect was observed. Namely, overall ANOVA on **haptic-E2** revealed that the two groups differed in height estimates in bi-modal conditions, i.e., in presence of sound. Having found this effect, we further examined the different distributions in the results between musicians and non-musicians, by analyzing over- and under-estimations of perceptual haptic errors in the bi-modal conditions (see histograms in Figure 8 and in Figure 9). Two main trends can be observed in these histograms. First, the estimates of non-musicians tended to be more precise in the *pitch 1* condition (see Figure 8). Second, musicians exhibited a peculiar behavior in the *random pitch* condition, with a tendency in over-estimating especially larger heights (see Figure 9).

For the musicians group, the generic significant effect of sound, further qualitatively analyzed, is in accordance with previous literature, which suggests that musicians are more sensitive than non-musicians to the spatial association between pitch and height (e.g., Rusconi et al., 2006). Regarding the interpretation of the specific results discussed above, we can hypothesize that non-musicians perceived the information provided by the *pitch1* and *pitch2* conditions as coherent with height increments but made no actual use of pitch information, resulting in similarities with uni-modal condition.

On the other hand, significant effect of condition for musicians, together with their unexpected behavior (tendency to over-estimation) in the *random pitch* condition may suggest that musicians exploited auditory information in a different way than the one initially hypothesized. The *random pitch* condition differ from the two informative bimodal conditions in that it provides an auditory feedback with a much denser musical texture: since random pitches are generated at every cm in heights, active exploration of the step produces a large number of different pitches in time, or notes-per-second (NPS), as opposed to the continuous sweep produced in the *pitch1* and *pitch2* conditions. The NPS feature is in fact often employed in music content classification and is associated to energetic properties of the musical signal (e.g., Mion and De Poli, 2008). The tendency to over-estimation in the *random pitch* condition may therefore be a consequence of the more energetic character of auditory feedback, which musicians associated to higher step-riser heights.

This latter explanation is clearly speculative. However, it enables to introduce a final remark. The results of the current study suggests that the association between pitch and height is too subtle to affect estimates gathered by a robust sense like touch. Perhaps, other acoustic and musical parameters (loudness, spectral centroid, spectral cues, notes-per-second, etc.) may have a stronger effect and should be analyzed in further experiments.

### Conflict of interest statement

The authors declare that the research was conducted in the absence of any commercial or financial relationships that could be construed as a potential conflict of interest.

## Footnotes

^1^A 6-DOF (degrees of freedom) position input and 3-DOF force output device with a stylus grip (see http://www.sensable.com for technical specifications).

^2^http://www.h3dapi.org/.

^3^http://www.web3d.org/x3d/.

^4^http://puredata.info/.

^5^No equal heights were presented also between the last trial in the 1st block and first trial in the 2nd block.

^6^We observed violations in a small number of these preliminary tests. However, because these tests are performed to limit the type I error and the current study reports many non-significant results (for example, we did not observe the hypothesized modulation of haptic height perception by musical pitch), these small violations do not influence the conclusions of the study.

^7^Since haptic estimations in uni-modal condition (silence) were taken as reference for the calculation of E3, only bi-modal conditions were considered.
